# Geographic Drivers of Antimicrobial Use and Resistance in Pigs in Khon Kaen Province, Thailand

**DOI:** 10.3389/fvets.2021.659051

**Published:** 2021-04-28

**Authors:** Laura Huber, Gunilla Ström Hallenberg, Kamonwan Lunha, Thongpan Leangapichart, Jatesada Jiwakanon, Rachel A. Hickman, Ulf Magnusson, Marianne Sunde, Josef D. Järhult, Thomas P. Van Boeckel

**Affiliations:** ^1^Department of Pathobiology, College of Veterinary Medicine, Auburn University, Auburn, AL, United States; ^2^Health Geography and Policy Group, Institute of Environmental Decisions, ETH Zürich, Zürich, Switzerland; ^3^Department of Clinical Sciences, Swedish University of Agricultural Sciences, Uppsala, Sweden; ^4^Section for Food Safety and AMR, Norwegian Veterinary Institute, Oslo, Norway; ^5^Research Group Preventive Technology Livestock, Khon Kaen University, Khon Kaen, Thailand; ^6^Department of Medical Sciences, Uppsala University, Uppsala, Sweden; ^7^Center for Diseases Dynamics Economics & Policy, Washington, DC, United States

**Keywords:** *Escherichia coli*, antimicrobial resistance, antimicrobial use, pig, intensive production

## Abstract

In Thailand, pig production has increased considerably in the last decades to meet a growing demand for pork. Antimicrobials are used routinely in intensive pig production to treat infections and increase productivity. However, the use of antimicrobials also contributes to the rise of antimicrobial resistance with potential consequences for animal and human health. Here, we quantify the association between antimicrobial use and resistance rates in extensive and intensive farms with a focus on geographic proximity between farm and drugstores. Of the 164 enrolled farms, 79% reported using antimicrobials for disease prevention, treatment, or as a feed additive. Antimicrobial-resistant *E. coli* were present in 63% of farms. These drugs included critically important antimicrobials, such as quinolones and penicillins. Medium-scale farms with intensive animal production practices showed higher resistance rates than small-scale farms with extensive practices. Farms with drug-resistant *Escherichia coli* were located closer to drugstores and a had a higher proportion of disease than farms without drug-resistant *E. coli*. We found no association between the presence of resistance in humans and antimicrobial use in pigs. Our findings call for actions to improve herd health to reduce the need for antimicrobials and systematic training of veterinarians and drugstore owners on judicious use of antimicrobials in animals to mitigate resistance.

## Introduction

Antimicrobial resistance (AMR) is a threat to the long-term viability of the animal production sector and potentially to human health (1). AMR is aggravated by the consumption of antimicrobials in animals, which represents 73% of all antimicrobial sales globally (2). In pig production systems, antimicrobials are used routinely for the prevention and treatment of diseases and, in some countries, as growth promoters (3, 4). The increase in demand for animal protein worldwide has led to rapid intensification of animal production (5, 6). As a consequence, antimicrobial use has increased considerably, leading to a rise of AMR prevalence in animals and humans worldwide (7–10). Due to the concern of zoonotic transmission of AMR from animals to humans (11), this has important consequences for human health and also for the long-term viability of the production of animals.

Countries transitioning toward intensive production, such as Thailand (12), may be of particular concern for the spread of animal diseases. In these countries, extensive and intensive animal production farms coexist (13), and this situation may favor the spread of AMR (14). In these countries, resources are limited, and investments in improvement of biosecurity may lag behind the rapid transition from small- to large-scale pig production systems (15). In Asia, control of infectious diseases is often based on the use of antimicrobials without appropriate supervision by veterinarians (16, 17). As a consequence of poorly implemented and unregulated development of the pig production sector, it is suggested that a transitioning system may have an increased reliance on antimicrobials to control animal disease (16, 18). In transitioning countries, monitoring antimicrobial use (AMU) is, thus, crucial to anticipate the impact of intensification on AMR trends globally.

Previous studies show a correlation between pig production systems (i.e., intensive versus extensive) and disease prevalence (19, 20). Small-scale farms with extensive pig production are characterized by low biosecurity levels and frequent contact between animals, humans, and wildlife. In large-scale farms with intensive pig production, animals share a small space, and the farm produces a large amount of manure, resulting in increased disease transmission between animals (16, 19). In Thailand, farm-level characteristics, such as pig and farm density, were found to be an important risk factor for several diseases (12, 21, 22). Studies are lacking, however, to address the link between prevalence of herd diseases, the consequent increase of AMU against these diseases, and levels of AMR.

Mapping AMR rates is an important tool to plan interventions against AMR and encourage AMU stewardship in regions most critically affected (23). Large-scale geographic analyses have mapped resistance prevalence globally (9) and regionally (Zhao et al., unpublished data.). However, thus far, few studies have attempted to understand the fine-scale effects of distance and geographic contiguity on the spread of AMR. The identification of fine-scale AMR hot spots could enable coordinated local actions to mitigate AMR.

In the Khon Kaen province of Thailand, pig production farms are most commonly family-owned, small-scale farms (with fewer than 50 sows) (24); however, the occurrence of company-owned, medium-scale commercial farms (with 100 to 500 sows) is progressively increasing. This study addresses if farm-level factors that differ between small- and medium-scale farms—such as the number of animals per farm, use of antimicrobials added in the feed, presence of herd disease, AMU—have contributed to the rise of AMR in pigs, farmers, and people in contact with farmers in the Khon Kaen province of Thailand. We enrolled 164 pig farms-−51 medium-scale intensive farms and 113 small-scale extensive farms—in Khon Kaen province and collected fecal samples from pigs and humans (with direct or indirect contact with the pig production) and conducted surveys on factors potentially associated with AMR on farms. The specific aims were to (i) identify the factors associated with AMR in *Escherichia coli* in pigs and humans, (ii) map the geographic distribution of drug-resistant *E. coli* in pig and human samples in the Khon Kaen province, and (iii) quantify the relationship between AMR levels and geographic proximity between farms.

## Materials and Methods

### Samples and Survey Collection

We classified farms included in this study as small- or medium-scale farms according to the number of sows (small-scale included farms with <50 sows/farm and medium-scale included farms with 100 to 500 sows/farm). The small-scale farms were family owned, and for the majority of the farmers, keeping pigs was not their main income; these farms were then regarded as extensive (24). In contrast, the medium-scale farms were company owned with standard protocols for medication, vaccination, and feed; these farms were, thus, classified as intensive (24). In this cross-sectional study, 164 pig farms located in Khon Kaen province in Thailand were enrolled, including 113 classified as small-scale farms and 51 medium-scale farms. All medium-scale farms from two districts (*n* = 51) were selected, and an equal number of small-scale farms was included from the same two districts. The small-scale farms selected were located the closest to medium-scale farms. The remaining 62 small-scale farms were included by convenience sampling in six surrounding districts without medium-scale farms.

Between September and December 2018, fecal samples were collected by rectal swabs from 1–10 sows (mean ± SD, 5 ± 3.5) per small-scale farm and 10 per medium-scale farm either by the employed field veterinarian or by research assistants (14). Fecal samples were also collected from humans in direct contact with the pig production and from humans sharing the household but without direct contact with the pigs. Surveys (24) were conducted at all farms about farm size, level of education of the farmers, number of animals at the farm, feed management, presence of herd disease, methods for disease prevention, AMU (including use of antimicrobials in the farm and total cost), the responsible professional giving instructions for AMU (in small-scale farms), and manure management.

### Sample Processing

*E. coli* isolates recovered from fecal samples were subjected to susceptibility testing using disc diffusion for seven antimicrobials: cefotaxime, ciprofloxacin, gentamicin, chloramphenicol, meropenem, tetracycline, and trimethoprim/sulfamethoxazole. Antimicrobial susceptibility testing results are published elsewhere (14). EUCAST guidelines were followed (25), using *E. coli* CCUG 17620 as control.

### Statistical Analysis

In this study, susceptibility testing results were recorded at the farm level and not the isolate level. For a given farm, three *E. coli* isolates were randomly selected from each host category (humans, contact humans, and no-contact humans) and susceptibility testing fsor seven antimicrobials was performed. The proportion of isolates resistant to each of the antimicrobials was then recorded and used in this analysis. Throughout the manuscript, the number of antimicrobials presenting at least one resistant *E. coli* isolate in pigs and contact and non-contact human samples are assigned as R_pig_, R_contact_, and R_no−contact_, respectively. Thus, for each farm, R_pig_ ≥ 3 means that antimicrobial-resistant *E. coli* was found in ≥ 3 antimicrobials tested.

AMU was estimated by dividing the total amount of money used on antimicrobials [recorded in Thai Baht (THB), and reported in this paper as U.S. dollars (1 USD = 31.5 THB)] by the total number of pigs in each farm.

We compared farms with R_pig_, R_contact_, or R_no−contact_ ≥ 3 to farms with R_pig_, R_contact_, or R_no−contact_ < 3 in regard to number of sows, AMU, and minimal distance between farms and drugstores (in Km). Moreover, comparisons of median R_pig_, R_contact_, and R_no−contact_ were done between farm size, farms that presented disease in pigs or not, that used antimicrobial added in the feed or not, and categories of manure management (discarded, sold, used as fuel, used as fertilizer). For comparison between two group medians of independent samples, the Wilcoxon rank sum test was used. For more than two independent samples, Kruskal Wallis tests were used. Bonferroni correction of *p*-values were used when comparing multiple groups. To compare proportions between medium- and small-scale farms, a two-sample test for equality of proportions with continuity correction was used.

For the evaluation of the effect of farm size, management factors, AMU, and presence of resistance in pigs and humans on resistance levels (R_pig_, R_contact_, and R_no−contact_), least absolute shrinkage and selection operator (LASSO) regression was used to select variables to be included in the generalized linear models. Cross-validation was done by dividing the data set into three sets. Using the variables included by the LASSO regression and their interactions terms, binomial generalized linear models were tested. The best model fit was selected by backward selection, removing from the model the least significant variables. The model fits were compared using the Akaike information criteria and the likelihood ratio test.

### Ethical Approval

The study was approved by the Khon Kaen University Ethics Committee (Project ID: HE612268 and 0514.1.75/66 respectively). Informed consent was obtained from each human subject. Permission of the owner was obtained to collect samples from pigs.

## Results

### Demography of Pigs and Sows in the Enrolled Farms

Small-scale farms held a median of 19 pigs per farm (IQR, 10–36 pigs per farm) and three sows per farm (IQR, 2–5 sows per farm), and medium-scale farms held a median of 156 pigs per farm (IQR, 428–594 pigs per farm) and 215 sows per farm (IQR, 153–239 sows per farm).

### Use of Antimicrobials for Disease Prevention and Treatment on Farms

AMU for disease prevention and treatment occurred in 79.3% of the farms [130/164 farms (CI, 73.1–85.5%); Table 1]. The use of feed additive containing antimicrobials was reported in 15.2% of farms [25/164 (CI, 9.8–20.8%); Table 1].

**Table 1:** Number of farms, percentage, and 95% confidence interval (CI) from data collected from 164 farms (51 medium-scale farms and 113 small-scale farms) in Khon-Kaen, Thailand.

**Variable**	**Farm size**	**Yes (% and 95%CI)**	**No (% and 95%CI)**
Used drugs	Overall	130 (79.27; 73.06–85.47)	34 (20.73; 14.52–26.94)
	Medium	51 (100.00; 99.49–100.69)^*^	0 (0.00; −0.96–1.04)
	Small	79 (69.91; 61.46–78.37)	34 (30.09; 21.63–38.54)
Feed additive	Overall	25 (15.24; 9.74–20.75)	139 (84.76; 79.25–90.26)
	Medium	24 (47.06; 33.36–60.76)^*^	27 (52.94; 39.24–66.64)
	Small	1 (0.88; −0.88–2.61)	112 (99.11; 97.39–100.84)
Presence of disease	Overall	74 (45.12; 37.5–52.74)	90 (54.88; 47.26–62.49)
	Medium	48 (94.11; 87.66–100.58)^*^	3 (5.88; −0.58–12.34)
	Small	26 (23.0; 15.25–30.77)	87 (76.99; 69.23–84.75)
R_pig_ ≥ 3	Overall	104 (63.41; 56.04–70.79)	60 (36.58; 29.21–43.96)
	Medium	49 (96.07; 90.75–101.41)^*^	2 (3.92; −1.41–9.24)
	Small	55 (48.67; 39.46–57.89)	58 (51.32; 42.11–60.54)
R_contact_ ≥ 3	Overall	39 (23.78; 17.26–30.30)	125 (76.22; 69.70–82.74)
	Medium	12 (23.53; 11.89–35.17)	39 (76.47; 64.83–88.11)
	Small	27 (23.89; 16.03–31.75)	86 (76.11; 68.24–83.97)
R_no−contact_ ≥ 3	Overall	26 (15.85; 10.26–21.44)	138 (84.14; 78.56–89.74)
	Medium	8 (15.68; 5.71–25.67)	43 (84.31; 74.33–94.29)
	Small	18 (15.93; 9.18–22.67)	95 (84.07; 77.32–90.82)

* *P-value<0.05 by two-sample test for equality of proportions with continuity correction*.

Descriptions of other methods to prevent diseases used in the farms were reported previously by collaborators (24). These include vaccination (75% of small-scale farms vs. 100% of medium-scale farms), use of medicated feed (0.9% of small-scale farms vs. 47.1% of medium-scale farms), and use of fencing around the farm (14.2% of small-scale farms vs. 100% of medium-scale farms).

### Medium-Scale Farms Use More Antimicrobials and Have a Higher Proportion of Antimicrobial-Resistant *E. coli* in Pigs Than Small-Scale Farms

Medium-scale farms reported spending a significantly higher amount of money on antimicrobials than small-scale farms [median, 2.4 (range, 0.5–7.6) USD/pig vs. median, 1.4 (range, 0–15.9) USD/pig, *p*-value < 0.0001]. All medium-scale farms reported using antimicrobials to treat or prevent diseases compared with 69.9% [79/113 (CI, 61.5–78.4)] of the small-scale farms (Table 1). The proportion of medium-scale farms that use feed additive containing antimicrobials was also significantly higher than in small-scale farms [24/51 medium-scale farms; 47.1% (CI, 33.4–60.8) vs. 1/113 small-scale farms; 0.9% (CI, −0.9–2.6), *p-*value<0.001]. Moreover, a significantly higher proportion of medium-scale farms had herd disease compared with small-scale farms [48/51 medium-scale farms; 94.1% (CI, 90.8–101.4) vs. 55/113 small-scale farms; 48.7% (CI, 39.5–57.9), *p*-value < 0.0001].

**Figure 1:**
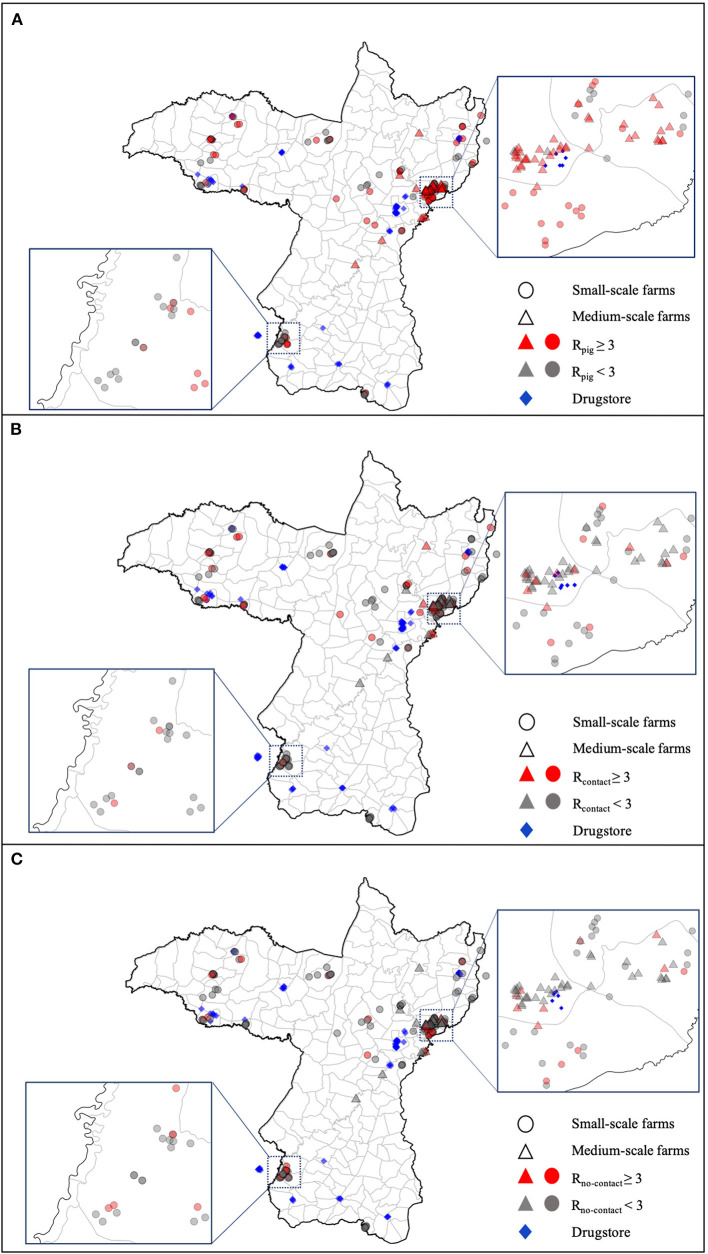
Geographic distribution of resistance in *E. coli* from **(A)** pigs, **(B)** contact humans, and **(C)** no-contact humans in Khon Kaen. Each data point represents a farm (*n* = 164). Farms with R_pig_, R_contact_, and R_no−contact_ ≥ 3 are represented in red, and farms with R_pig_, R_contact_, and R_no−contact_ < 3 are represented in gray. Triangles represent medium-scale farms, and circles represent small-scale farms. The location of the drugstores from which farms reported buying veterinary supplies are represented by the blue rectangles. Blue dashed squares show the zoomed view of the cluster regions.

The proportion of medium-scale farms with R_pig_ ≥ 3 was significantly higher than the proportion of small-scale farms with R_pig_ ≥ 3 [49/51; 96.1% (CI, 90.8–101.4%) vs. 55/113; 48.7% (39.5–57.9%), *p*-value < 0.0001; Table 1]. In pig samples, R_pig_ ≥ 3 was frequent for farms located in Northeast Khon Kaen, where a large number of medium-scale farms are located (Figure 1). In human samples, the R_contact_ ≥ 3 and R_no−contact_ ≥ 3 presents similar clustering as R_pig_ in Northeast Khon Kaen, however, with a lower proportion compared with R_pig_. Veterinary drugstore locations are geographically distributed in proximity with the pig farms, and at least six veterinary drugstores can be observed within the cluster of medium-scale farms in Northeast Khon Kaen (Figure 1). The average distance from small- to medium-scale farms did not differ between farms with R_pig_, R_contact_, and R_no−contact_ ≥ 3 and farms with R_pig_, R_contact_, and R_no−contact_ < 3 (*p*-values = 0.404, 0.226, and 0.252, respectively). R_pig_, R_contact_, and R_no−contact_ did not differ significantly between small-scale farms that are closer versus farther from medium-scale farms (*p*-values = 0.131, 0.923, and 0.368, respectively)

### Factors Associated With AMR in *E. coli*

**Figure 2:**
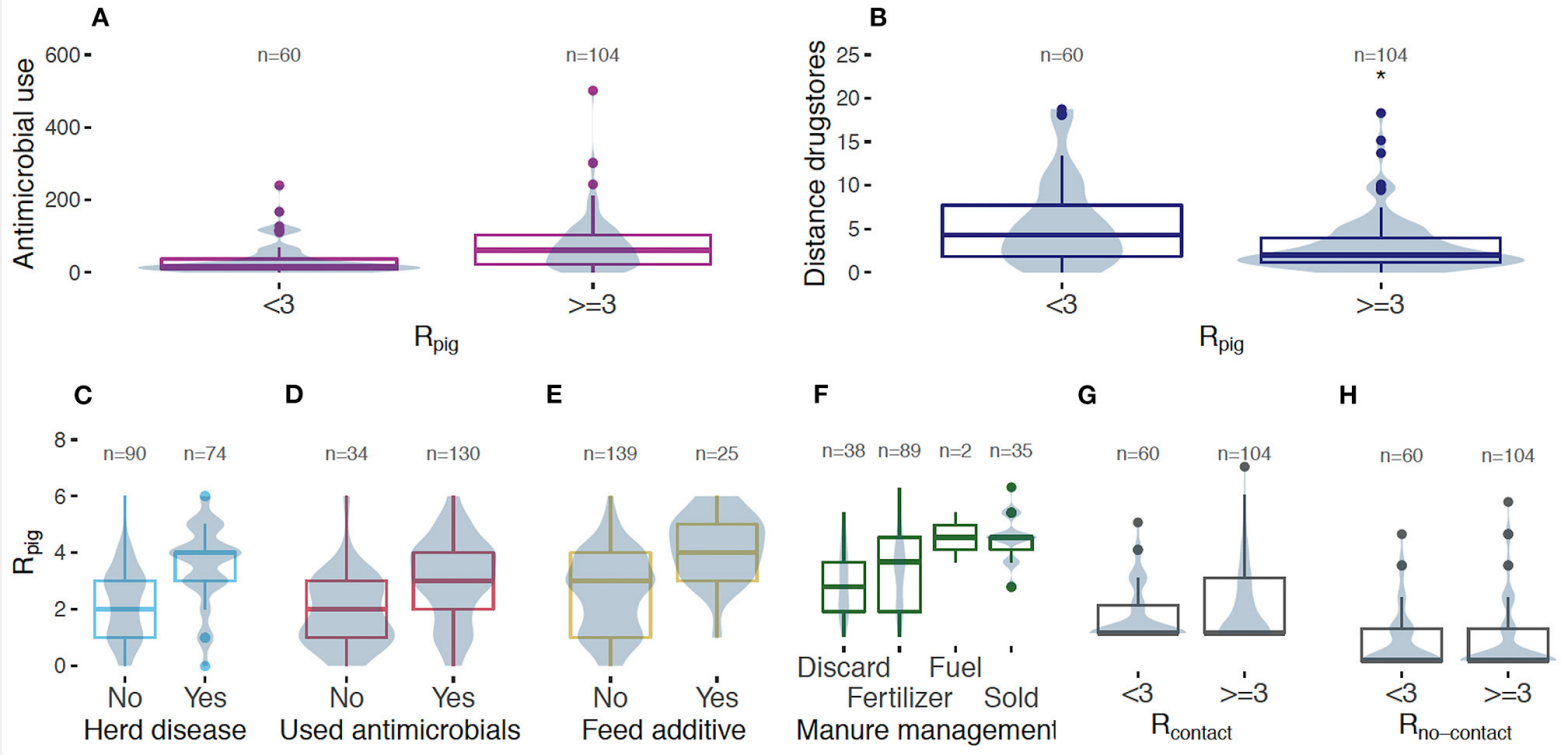
*E. coli* resistance in pig samples at the farm level in Khon Kaen, Thailand. Medians, interquartile range, minimum, and maximum of (i) the antimicrobial use (cost of antimicrobials per pig) **(A)** and the minimum distance (in Km) to drugstores **(B)** between farms that presented R_pig_ ≥ 3 or R_pig_ < 3; (ii) the R_pig_ between farms reporting herd disease or not **(C)**, between farms reporting to have used antimicrobials or not **(D)**, use of antimicrobials added in the feed or not **(E)**, between categories of manure management **(F)**, and between farms where R_contact_ **(G)** and R_no−contact_ **(H)** ≥ 3 or < 3. Distribution of the data points is represented by the violin plot in blue. Colored frames indicate that comparison between groups is significant with *p*-value < 0.05 by the Wilcoxon rank sum test or Kruskal Wallis test. Gray frames mean absence of significant differences.

Farms with R_pig_ ≥ 3 had significantly higher AMU and were located significantly closer to drugstores than farms with R_pig_ < 3 (*p*-values < 0.001; effect sizes of 0.378 and 0.284, respectively; Figure 2). Farms that reported herd disease and that used feed additive had significantly higher median R_pig_ than farms that did not (*p*-values < 0.001; effect size of 0.486 and 0.380, respectively). The median R_pig_ at farms where the manure is discarded and used as a fertilizer was significantly lower than in farms that sell manure (*p*-value < 0.001 for both; effect size of 0.596 and 0.410, respectively; Figure 2). The overall percentage of R_contact_ ≥ 3 was significantly higher than R_no−contact_ ≥ 3 [39/164; 23.78% (CI, 17.26–30.30%) vs. 26/164; 15.85% (CI, 10.26–21.44%); *p*-value = 0.048; Table 1]. No differences on the R_contact_ and R_no−contact_ between groups were found Supplementary Figures 1, 2.

### Medium-Scale Farms Have an Increased Likelihood of Antimicrobial-Resistant *E. coli* in Pigs

The best fitted LASSO-penalized generalized linear model using R_pig_ as an outcome variable shows that the size of the medium-scale farms is significantly and positively associated with resistance in pigs (Table 2). Unlike for animals, in humans, resistance levels could not be associated with risk factors (AMU in pigs, distance to drugstores, farm size).

**Table 2:** LASSO-penalized generalized linear model.

**Variable**	**Estimate**	**Standard error**	***P*-value**
AMU (money spent per pig)	0.00159	0.002482	0.522
Antimicrobial use (binary)	0.345485	0.454379	0.447
Shortest distance to drugstores (Km)	−0.024083	0.043337	0.578
Medium-scale farm	0.800876	0.392686	0.041^*^

*The outcome is R_pig_ in the farm level. Explanatory variables included antimicrobial use (AMU; continuous variable on the cost of antimicrobials per pig), use of antimicrobials (binary variable), minimal distance between farms and drugstores (continuous variable; in Km), and size of farms (binary variable; medium and small-scale farms)*.

* *P-value<0.05*.

### Small-Scale Farms Rarely Consult Veterinary Services Before Using Antimicrobials

Medium-scale farms included in this study are company owned, thus recommendations on antimicrobial indications are provided by veterinarians working for the company. In contrast, small-scale farms with extensive pig production are family-owned farms and do not always have access to veterinary assistance, seeking advice to treat and prevent diseases at drugstores (24).

The source of advice for using antimicrobials in animals was reported by 88 out of 113 small-scale farmers. From these, just 4/88 (4.6%) reported having consulted a veterinarian before using antimicrobials vs. 67/88 (76.1%) having obtained advice from veterinary drugstore owners. Out of 113 small-scale farms, 67 farms (59.3%) reported the most commonly used antimicrobial drugs, including enrofloxacin (26/67 farms; 38.8%), penicillin-streptomycin (15/67 farms; 22.4%), amoxycillin (10/67 farms; 14.9%), oxytetracycline (7/67 farms; 10.5%), gentamicin (3/67 farms; 4.5%), kanamycin (2/67 farms; 2.9%), chlorotetracycline (1/67 farm; 1.5%), linco-streptomycin (1/67 farm; 1.5%), oxytetracycline-streptomycin (1/67 farm; 1.5%), and penicillin (1/67 farm; 1.5%). In contrast, all medium-scale farmers (51/51 farms) reported seeking advice from veterinarians before using antimicrobials. The combination of penicillin and streptomycin was reported to be used in all medium-scale farms (51/51 farms).

## Discussion

We report resistant *E. coli* from pigs in 63% of the 164 enrolled farms in the province of Khon Kaen, Thailand. Antimicrobials are crucial for animal health and food safety in the animal production industry (4); however, their overuse has led to an increase of AMR (26). Globally, 120.88 million tons of pork are produced every year, representing 40% of total meat production (27). Pigs consume 32% of the overall veterinary antimicrobials sold in Europe (28). In our study, 79% of farms reported using antimicrobials for disease prevention and treatment, as feed additive, or both. These include antimicrobials considered as critically important to human health (29), such as streptomycin, fluroquinolones, and penicillins. The high proportion of resistant *E. coli* at farms calls for action to reduce the use of antimicrobials in this sector.

In this study, we found that intensive medium-scale farms used significantly more antimicrobials and had significantly higher AMR in pigs than extensive small-scale farms. Higher AMR levels in medium- compared with small-scale farms were observed previously (14). During the process of intensification of the food-animal sector, the risk of herd disease increases due to higher density of animals and of farms (19). Thus, during the intensification of production systems, the adoption of better biosecurity measures, nutrition, and health management are essential to prevent diseases, lowering the requirement for antimicrobial use (30–34). Our results show a significantly higher proportion of medium-scale farms reporting disease in pigs (94%) compared with the proportion of small-scale farms with disease in pigs (49%). Previously, collaborators indicated that significantly more medium-scale farms use vaccines and medicated feed to prevent diseases in pigs compared with small-scale farms (24). Taken together, our results suggest that implementation of better biosecurity measures to prevent diseases might not be following the rapid intensification of pig production in this region (12), resulting in higher use of antimicrobials in medium-scale farms and leading to the AMR rise. Based on these findings, measures to mitigate AMR in intensive medium-scale farms in Khon Kaen should focus on improving biosecurity. These measures can, in turn, reduce the routine use of antimicrobials (35, 36) and the prevalence of AMR.

Intensive medium-scale farms in Khon Kean are company owned; thus, recommendations on antimicrobial indications are provided by veterinarians working for the company. In contrast, extensive small-scale farms were found to receive advice on antimicrobial use from veterinary drugstores. Here, we found that farms with antimicrobial resistant *E. coli* in pigs were located closer to drugstores than farms without antimicrobial-resistant *E. coli*. Previous studies suggest that drugstore owners and their staff play an important role in antimicrobial stewardship (37, 38). Therefore, differently from the medium-scale farms, effective intervention scenarios to reduce antimicrobial use in small-scale farms should focus on (i) facilitating and stimulating the access of farmers to animal health workers and (ii) implementing disease diagnostic services in drugstores. Moreover, a recent code of practice for veterinary drug use by the Ministry of Agriculture and Cooperatives in Thailand requires veterinarian supervision for antimicrobial sales at veterinary drugstores (39). Thus, systematic training of veterinarians and drugstore staff on judicious use of antimicrobials is needed to reduce antimicrobial use. Previous works have shown that, in some regions of Europe, farmers are eager to reduce AMU and also expressed concerns about the feasibility and efficiency of such measures (40). The conditions faced in Europe might not be comparable with the ones in an emerging economy such as Thailand. However, it suggests that reducing the heavy reliance on antimicrobial use depends on systematic education and training of veterinarians, farmers, and drugstore owners on the judicious use of antimicrobials.

In this study, AMR in pigs and humans was found in a large number of farms in Khon Kaen province. Because medium-scale farms had a significantly higher proportion of AMR in pigs than small-scale farms, AMR is clustered in the region where medium-scale farms are located (Northeast Khon Kaen). However, no associations were found between farms that had resistance in pigs or humans in relation to the distance to other farms. Moreover, the proximity of small- to medium-scale farms did not influence the proportion of resistance in pigs or humans. Therefore, in this study, geographical distance does not seem to play an important role in resistance patterns. Despite this lack of evidence that the location of farms influences resistance levels, we found that pigs at medium-scale farms carried resistant *E. coli* against antimicrobials that were not reported to be used in these farms. Thus, resistant *E. coli* could have spread from farm to farm through the movement of humans, animals, and products. It was previously suggested that resistant *Enterobacteriaceae*-carrying resistance genes can spread over large geographic distances (41). These isolates have contributed to the spread of multidrug resistances through the sharing of plasmids and resistance genes among Gram-negative bacteria (42). Therefore, these results indicate that antimicrobial administration practices used in a group of farms might increase AMR levels in animals at nearby farms in Khon Kaen. This raises awareness of the importance of tackling AMR in both intensive medium-scale and extensive small-scale farms due to the risk of AMR transmission across space. Alternatively, the use of antimicrobials in medium-scale farms might have been underreported. Moreover, the acquisition of multidrug-resistant genetic mobile elements (43) combined with the use of penicillin and streptomycin at these farms might have co-selected for multidrug-resistant *E. coli* at these farms.

The transmission of resistant bacteria from livestock to humans is of public health concern (11). Here, we found that humans in direct contact with pigs had significantly higher levels of resistance than humans without direct contact with pigs (e.g., family members of farmers). However, no associations were found between AMR in pigs and humans. Thus, further analysis is needed to understand the risk of AMR in humans originating from pigs in Khon Kaen. Previously, whole-genome phylogenetic analysis of *Clostridium difficile* strains from 22 countries revealed extensive co-clustering of human and animal strains, indicating a highly linked intercontinental transmission network between humans and animals (44). Similarly, through future studies using phylogeographic analysis, the association of the risk factors (such as density of animals and AMU) with transition frequencies among farms and hosts in Khon Kaen could be accessed (45).

The results from this work bring important information about the effect of AMU on AMR in Khon Kaen. However, AMU in pigs at these farms was estimated based on the total amount of money spent on antimicrobials reported by farmers (at small-scale farms) or by the company (at medium-scale farms). Thus, this estimation could be biased by price fluctuation of each antimicrobial and differences in price between bulk antimicrobial purchases at medium-scale farms vs. individual purchases at small-scale farms. Moreover, antimicrobial use might have been underreported, especially by small-scale farmers, with which its use was frequently not supervised by a veterinarian. For example, the use of antimicrobial via feed might have been used to a larger extent in small-scale farms unknowingly. In this study, we have not explored the association between proportion of AMR and method of manure management because farm size might be a confounder. We did, however, control for the effect of manure management and its interactions with other variables in our model selection procedure.

The results from this study call for measures to mitigate AMR to critically important antimicrobials. AMR in pigs was frequent in Northeast Khon Kaen, where a large number of medium-scale farms are located. Although our findings suggest that geographic proximity is not a clear driver of the spread of AMR, it is possible that resistance has spread between farms in Khon Kaen due to the movement of animals and people. Our results show the importance of tackling AMR in both small- and medium-scale farms to avoid transmission of AMR across space. This study suggests that implementation of good biosecurity measures to prevent diseases might not be following the rapid intensification of pig production in this region. Thus, it is important to promote herd health by improving biosecurity and, thus, reduce prevalence of diseases and the need to use antimicrobials.

## Data Availability Statement

The raw data supporting the conclusions of this article will be made available by the authors, without undue reservation.

## Ethics Statement

The studies involving human participants were reviewed and approved by Khon Kaen University Ethics Committee (Project ID: HE612268 and 0514.1.75/66 respectively). The patients/participants provided their written informed consent to participate in this study. The animal study was reviewed and approved by Khon Kaen University Ethics Committee (Project ID: HE612268 and 0514.1.75/66 respectively).

## Author Contributions

LH data manipulation and analysis, scientific report writing, and editing. TV study design, supervision, and scientific editing. GH, TL, and JJ study design, data collection, and scientific report editing. RH, JDJ, and MS study design and scientific report editing. UM study design, supervision, coordination, and scientific editing. All authors contributed to the article and approved the submitted version.

## Conflict of Interest

The authors declare that the research was conducted in the absence of any commercial or financial relationships that could be construed as a potential conflict of interest.

## Abbreviations

AMR: antimicrobial resistance
AMU: antimicrobial use
THB: Thai Baht
LASSO: least absolute shrinkage and selection operator.
