# Supplementary material for: Geographic Drivers of Antimicrobial Use and Resistance in Pigs in Khon Kaen Province, Thailand
Source: Front Vet Sci. 2021 Apr 28;8:659051. doi: 10.3389/fvets.2021.659051 (PMC8113701; doi:10.3389/fvets.2021.659051)
Supplement: Supplementary file 1 [file Data_Sheet_1.pdf]

Supplementary material

Open data access:

https://github.com/laurahuber/Data_Analysis

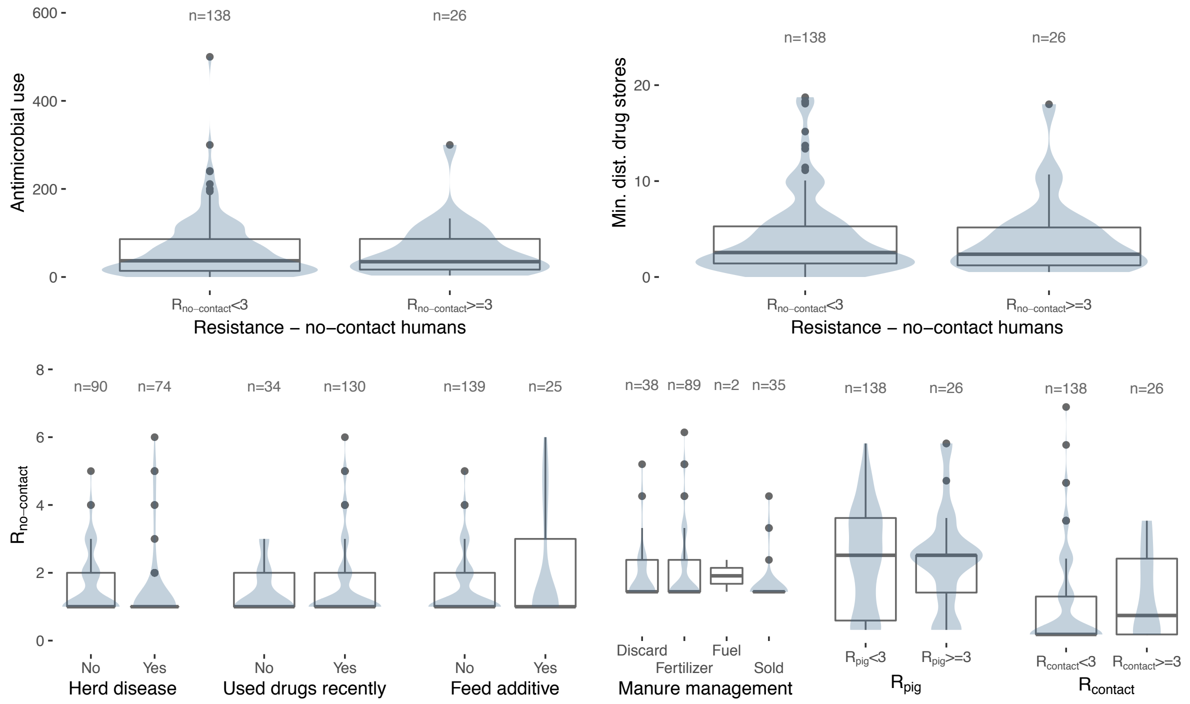

Supplementary figure 1. ***E. coli*** **resistance in contact-human samples in the farm level.** Medians, Interquartile Range, minimum, and maximum of (i) the AMU (THB/pig) and the minimum distance to drugstores, between farms that presented $R_{contact} \geq 3$ or $R_{contact} < 3$; (ii) the $R_{contact}$ between farms where R_{pig} and $R_{no\text{-}contact} \geq 3$ or < 3, between farms reporting pig disease or not, between farms reporting to have used antimicrobials or not, use of feed additive or not, and between categories of manure management. Distribution of the datapoints are represented by the violin plot in blue. No statistically significant differences were found between groups with significance set at 5% by the Wilcoxon rank Sum test or Kruskal Wallis test.

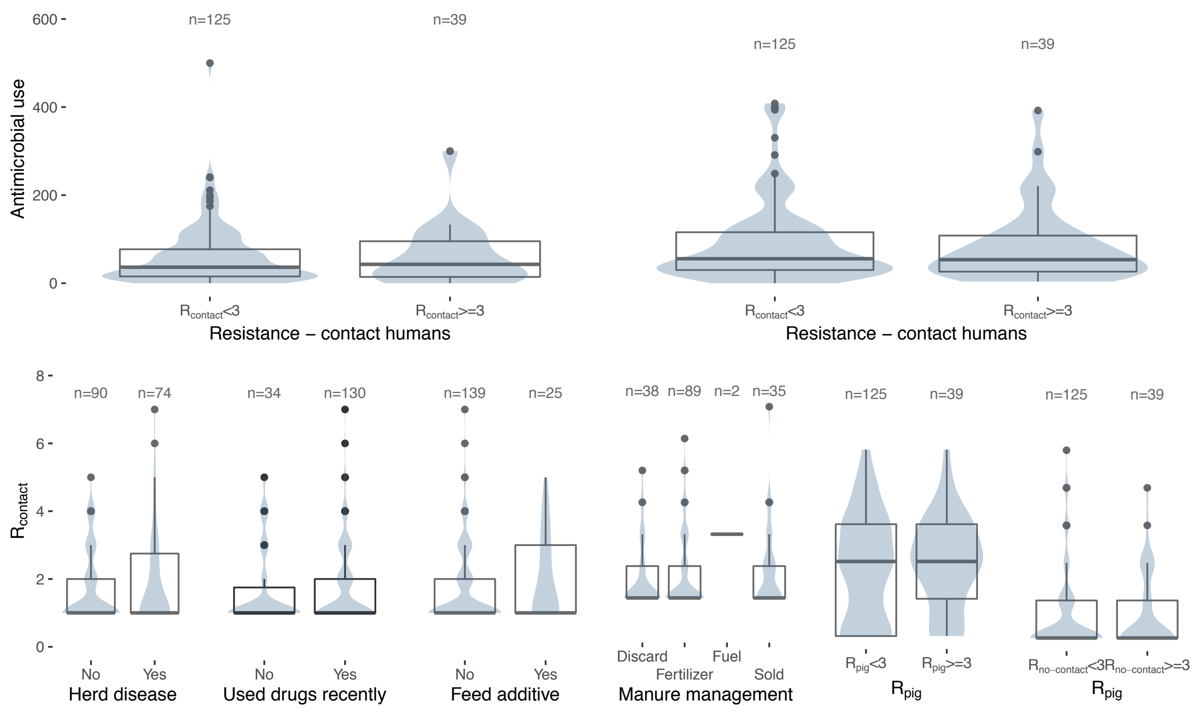

Supplementary figure 2. ***E. coli*** **resistance in no contact-human samples**. Medians, Interquartile Range, minimum, and maximum of (i) the AMU (THB/pig) and the minimum distance to drugstores, between farms that presented $R_{no\text{-}contact} \geq 3$ or $R_{no\text{-}contact} < 3$; (ii) the $R_{no\text{-}contact}$ between farms where R_{pig} and $R_{contact} \geq 3$ or < 3, between farms reporting herd disease or not, between farms reporting to have used antimicrobials or not, use of feed additive or not, and between categories of manure management. Distribution of the datapoints are represented by the violin plot in blue. No statistically significant differences were found between groups with significance set at 5% by the Wilcoxon rank Sum test or Kruskal Wallis test.

Model validation

For model validation, a more robust analysis was conducted using the best fitted LASSO-penalized generalized linear model (Table 1). The binary outcome was obtained by comparing the proportion of R_{pig} in the farm level with a vector with randomly created values from 0-1. Over 10 interactions, when proportion of R_{pig} was higher than the random vector

value, the number 1 was assigned. Similarly, when proportion of R_{pig} was lower than the random vector value, the number 0 was assigned. The mean and 95% CI's for the coefficients, Standard Error (SE), Z values, and p-values obtained from 10 interactions is shown in Supplementary Table 2.

The bootstrapped LASSO-penalized generalized logistic regression model, medium-scale farm was positively and significantly associated with R_{pig} (Supplementary Table 2; Supplementary Figure 3). Farms that reported using antimicrobials was positively associated with R_{pig} and significance level 95% CI includes p-value<0.05 (Supplementary Table 2; Supplementary Figure 3).

Supplementary Table 1. Bootstrapped output from LASSO-penalized generalized logistic regression. The binary outcome was obtained by comparing the R_{pig} in the farm level with a vector of randomly created values from (0-1) in 10 interactions. Explanatory variables included antimicrobial use (AMU; continuous variable on the cost with antimicrobials by number of pigs), use of antimicrobials (binary variable), shortest distance between farms and drug-stores (continuous variable), and size of farms (binary variable; medium- and small-scale farms). The mean and 95% CI's are presented for the coefficients, Standard Error (SE), and p-values obtained from 10 interactions.

Variable	Coefficients	Standard Error	p-value
Intercept	-1.94 (1.04, -0.84)	0.21 (0.20, 0.21)	5.17e-05 (-1.83e-05, 0.0001)*
AMU (cost/number of pigs)	0.001 (0.001, 0.001)	0.001 (0.001, 0.001)	0.33 (0.13, 0.52)
Used antimicrobials	0.33 (0.17, 0.48)	0.20 (0.20, 0.21)	0.27 (0.02*, 0.53)

Medium-scale farms	0.86 (0.75, 0.98)	0.18 (0.18, 0.18)	2.38e-05 (-9.16e-07 4.85e-05)*
Min. dist. drug stores (Km)	-0.021 (-0.033, 0.009)	0.019 (0.019, 0.02)	0.39 (0.31, 0.65)

* p-value<0.05

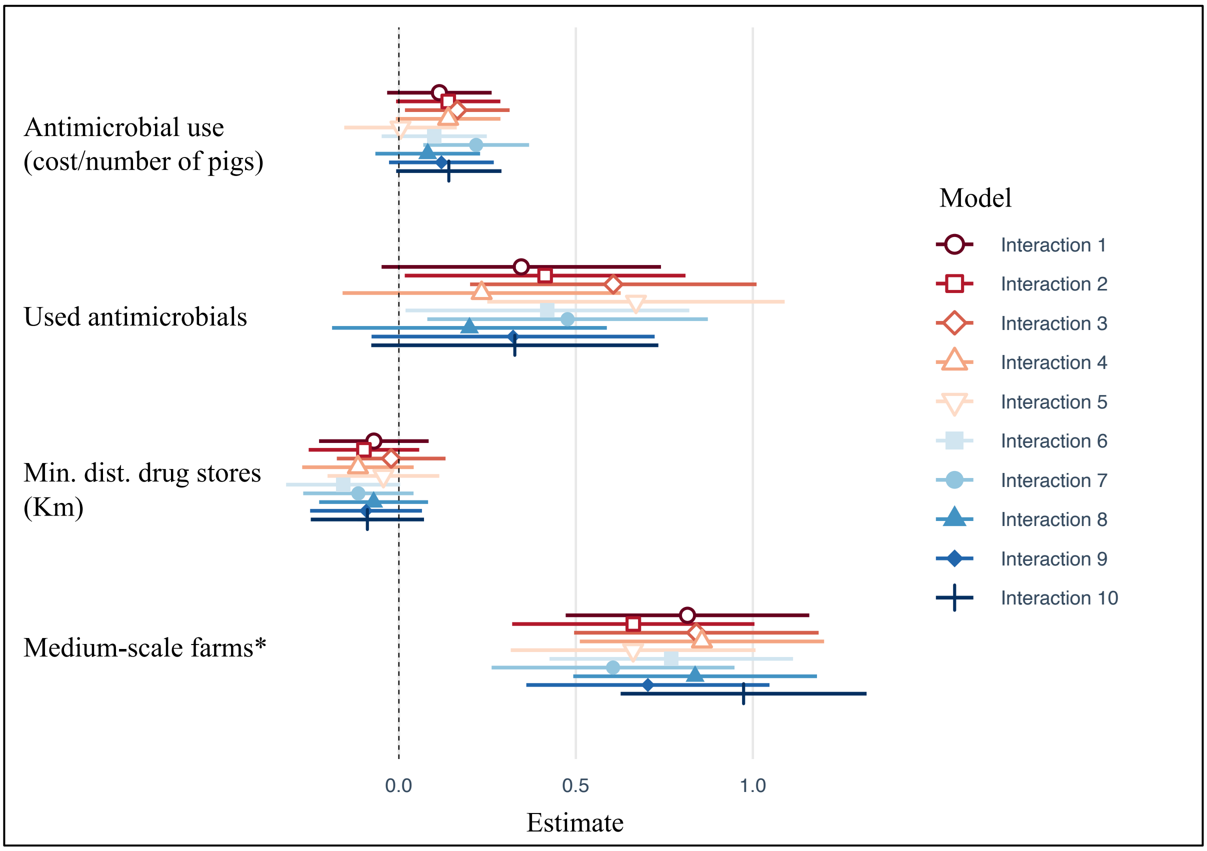

Supplementary figure 3. Bootstrapped output from 10 interactions of LASSO-penalized generalized logistic regression. The binary outcome was obtained by comparing the R_{pig} in the farm level with a vector of randomly created values from (0-1). Explanatory variables included antimicrobial use (continuous variable on the cost with antimicrobials by number of pigs), use of antimicrobials (binary variable), minimal distance between farms and drugstores (continuous variable), and size of farms (binary variable; medium- and small-scale farms). *p-value (and 95% CI) <0.05.
